# Multimodal Impact of Number of Metastases and Genetic Alterations on Survival in Metastatic Non–Small Cell Lung Cancer

**DOI:** 10.1200/PO-25-00597

**Published:** 2026-05-14

**Authors:** N. Ari Wijetunga, Nikhil P. Mankuzhy, Emily S. Lebow, Lillian A. Boe, Luke R.G. Pike, Adam J. Schoenfeld, Narek Shaverdian, Tafadzwa L. Chaunzwa, David R. Jones, Jamie E. Chaft, Charles M. Rudin, Mark G. Kris, Helena A. Yu, Jason C. Chang, Puneeth Iyengar, Daniel R. Gomez

**Affiliations:** ^1^ Department of Radiation Oncology, Memorial Sloan Kettering Cancer Center, New York, NY; ^2^ Department of Radiation Oncology, University of North Carolina, Chapel Hill, NC; ^3^ Department of Radiation Oncology, University of Pennsylvania, Philadelphia, PA; ^4^ Department of Epidemiology and Biostatistics, Memorial Sloan Kettering Cancer Center, New York, NY; ^5^ Department of Medicine, Thoracic Oncology Service, Memorial Sloan Kettering Cancer Center, New York, NY; ^6^ Department of Surgery, Thoracic Surgery Service, Memorial Sloan Kettering Cancer Center, New York, NY; ^7^ Department of Pathology, Memorial Sloan Kettering Cancer Center, New York, NY

## Abstract

**PURPOSE:**

Oligometastatic disease (OMD) is an intermediate state of metastatic disease in which metastasis-directed therapy (MDT) may improve outcomes. The classification of OMD is inconsistent, typically defined by number of metastases without considering tumor biologic characteristics. To help optimize patient selection for MDT, we analyzed integrated genomic sequencing results from patients with metastatic non–small cell lung cancer (NSCLC).

**MATERIALS AND METHODS:**

Patients with metastatic NSCLC who had molecular sequencing through a validated institutional assay (MSK-IMPACT) were included. The number and location of metastases were manually annotated (assigned 1-10 and >10). Individual gene variant scores were analyzed by gene length and patient mutation burden. Analysis was completed in R and included maximally selected rank statistics, agglomerative hierarchical clustering, and the chi-square test for independence.

**RESULTS:**

In total, 844 patients had clinical data, tissue sequencing, and annotated imaging for analysis. Of these, 635 had >10 metastases (75.2%), and 209 had 1-10 metastases (24.8%). The cutpoint that maximized overall survival (OS) was four metastases, and six mutation signatures were identified. For patients with 1-4 metastases, *TERT* and *KMT2D* had inferior OS, while for those with ≥5 metastases, *EGFR*, *ALK*, and *TBX3* had superior OS.

**CONCLUSION:**

The cutpoint that maximized difference in OS was four metastases, but incorporating genetic alteration information modified this criterion. These findings were proof of principle that integrating multimodal data beyond number of lesions can better identify patients with metastatic NSCLC who may be candidates for MDT.

## INTRODUCTION

Metastatic non–small cell lung cancer (NSCLC) has traditionally been treated as a single disease with poor outcomes.^1^ However, molecularly targeted agents and immunotherapy for disease without oncogenic mutations have shifted treatment paradigms toward precision oncology.^2^ Oligometastatic disease (OMD) describes an intermediate state of metastatic disease characterized by limited metastases and a more favorable prognosis.^3,4^ Along with effective targeted therapy and immunotherapy,^5-8^ randomized clinical trials have demonstrated improved outcomes in OMD with consolidative metastasis-directed therapy (MDT), such as surgery or radiotherapy.^9-13^

**CONTEXT**

**Key Objective**
Identifying patients with metastatic non–small cell lung cancer (NSCLC) who would benefit from metastasis-directed therapy (MDT) remains a challenge, as common definitions of oligometastatic disease (OMD) rely on counting metastases at a static time point. We aimed to assess how genomic alterations can correlate with overall survival (OS) when added to number of metastases.
**Knowledge Generated**
The numerical cutpoint for separating OS based on number of metastases was 4; however, the presence of certain genomic alterations changed this cutpoint. There is heterogeneity in genomic alterations present at metastases diagnosis when categorizing patients by number of metastases into 1-4, 5-10, and >10 cohorts, and they differentially associate with survival depending on the cohort.
**Relevance**
Genomic data should be considered when selecting patients with metastatic NSCLC for MDT, and these results provide evidence that integrating multimodal data to number of metastases may better identify patients with OMD.

Substantial heterogeneity is present in patient selection for benefit from comprehensive MDT.^14,15^ Estimating incidence of OMD and extrapolating results between studies remain challenging.^16^ Ongoing trials, such as SABR-COMET-10, are investigating MDT to up to 10 metastasis.^16^ Overall, defining OMD by metastases enumeration at a single time point has been inconsistent and arbitrary, and may not reflect disease trajectories. These limitations likely contributed to the negative results of a recent national phase II/III trial investigating MDT for oligometastatic NSCLC.^17^ Incorporating the biological phenotype may better inform patient selection for MDT.^18,19^

In this study, we used a comprehensive institutional database with clinical, imaging, and genomic information to identify patients with metastatic NSCLC with a broad range of metastatic burden at diagnosis. Our objectives were to estimate the frequency of OMD, examine the prognostic influence of metastasis number and tumor genetics with overall survival (OS), and inform selection for comprehensive MDT. We hypothesized that combining tumor genetics with metastases number at diagnosis would better delineate disease trajectories and enhance the classification of OMD.

## MATERIALS AND METHODS

### Clinical Database

After institutional review board approval with a waiver of informed consent, we retrospectively identified patients from a single-institution database diagnosed with metastatic NSCLC from 2012 to 2020. Inclusion criteria were patients age 18 years or older who (1) were diagnosed with metastatic NSCLC, including both de novo metastases or metastatic recurrence after prior definitive local therapy, (2) underwent targeted exome sequencing of tissue from a primary or metastatic tumor at metastasis diagnosis using MSK-IMPACT (Integrated Mutation Profiling of Actionable Cancer Targets),^20^ and (3) had imaging (positron emission tomography-computed tomography [PET-CT], CT, and magnetic resonance imaging) within 30 days of metastasis diagnosis.

### Defining the Number of Metastases

We delineated extent of disease by counting the number of tumors or involved nodal stations (further described in Appendix 1) that would correspond with a clinically targetable region during MDT, consistent with prior randomized analyses.^8^ The presence of effusions, leptomeningeal disease, and other innumerable metastases classified patients as having >10 metastases as they represent a nontargetable treatment area for MDT.

### Correlating Metastatic Burden With OS

We then defined the cutpoint that maximized the difference in OS (estimated from metastatic diagnosis) in patients with 1-10 metastases using maximally selected rank statistics by examining all cutpoints between 1 and 10 metastases (Appendix 1).^21^ We performed internal validation of our cutpoint using a bootstrap resampling–based analysis with 1,000 bootstrap samples, each of which were drawn with replacement from the original data.

### Assessing Genetic Alterations Through MSK-IMPACT

All patients underwent MSK-IMPACT testing from primary or metastatic tissue obtained at the time of metastasis diagnosis (Table 1).^20^ For each patient, we identified altered genes, including both pathogenic variants and variants of unknown significance, using a previously published technique for desparsification of mutational data.^22^ Additional details are described in Appendix 1.

**TABLE 1.** Patient Data Stratified by Number of Metastases

Characteristic	1-10 Metastases			>10 Metastases	Total
	Overall (n = 209)	1-4 (n = 178)	5-10 (n = 31)	n = 635	N = 844
Number of involved organs, No. (%)					
1	123 (58.9)	120 (67.4)	3 (9.7)	121 (19.1)	244 (28.9)
2	62 (29.7)	50 (28.1)	12 (38.7)	169 (26.6)	231 (27.4)
3	15 (7.2)	8 (4.5)	7 (22.6)	159 (25.0)	174 (20.6)
4	7 (3.3)	0 (0)	7 (22.6)	100 (15.7)	107 (12.7)
5	1 (0.5)	0 (0)	1 (3.2)	54 (8.5)	55 (6.5)
6	1 (0.5)	0 (0)	1 (3.2)	25 (3.9)	26 (3.1)
7	0 (0)	0 (0)	0 (0)	6 (0.9)	6 (0.7)
9	0 (0)	0 (0)	0 (0)	1 (0.2)	1 (0.1)
De novo metastatic disease, No. (%)	190 (90.9)	159 (89.3)	31 (100)	599 (94.3)	789 (93.5)
Recurrent metastatic disease, No. (%)	19 (9.1)	19 (10.7)	0 (0)	36 (5.7)	55 (6.5)
Primary tumor histology, No. (%)					
Adenocarcinoma	163 (78.0)	141 (79.2)	22 (71.0)	527 (83.0)	690 (81.8)
NSCLC NOS	24 (11.5)	20 (11.2)	4 (12.9)	57 (9.0)	81 (9.6)
Other	1 (0.5)	1 (0.6)	0 (0)	7 (1.1)	8 (0.9)
Squamous cell carcinoma	21 (10.0)	16 (9.0)	5 (16.1)	44 (6.9)	65 (7.7)
Metastasis site of biopsy, No. (%)					
Adrenal gland	19 (9.1)	16 (9.0)	3 (9.7)	11 (1.7)	30 (3.6)
Bone	33 (15.8)	26 (14.6)	7 (22.6)	98 (15.4)	131 (15.5)
CNS	33 (15.8)	28 (15.7)	5 (16.1)	38 (6.0)	71 (8.4)
Liver	6 (2.9)	6 (3.4)	0 (0)	37 (5.8)	43 (5.1)
Lung/pleura	3 (1.4)	3 (1.7)	0 (0)	165 (26.0)	168 (19.9)
Lymph node	106 (50.7)	92 (51.7)	14 (45.2)	236 (37.2)	342 (40.5)
Muscle/soft tissue/skin	8 (3.8)	6 (3.4)	2 (6.5)	35 (5.5)	43 (5.1)
Other	1 (0.5)	1 (0.6)	0 (0)	15 (2.4)	16 (1.9)
Sex, No. (%)					
Female	116 (55.5)	96 (53.9)	20 (64.5)	363 (57.2)	479 (56.8)
Male	93 (44.5)	82 (46.1)	11 (35.5)	272 (42.8)	365 (43.2)
Age, years, median (IQR)	66 (59-73)	66 (59-73)	66 (60-70)	67 (59-74)	67 (59-74)
Race, No. (%)					
Asian	21 (10.0)	19 (10.7)	2 (6.5)	76 (12.0)	97 (11.5)
Black	11 (5.3)	8 (4.5)	3 (9.7)	41 (6.5)	52 (6.2)
Other	8 (3.8)	7 (3.9)	1 (3.2)	13 (2.0)	21 (2.5)
Unknown	7 (3.3)	5 (2.8)	2 (6.5)	23 (3.6)	30 (3.6)
White	162 (77.5)	139 (78.1)	23 (74.2)	482 (75.9)	644 (76.3)
Smoking, No. (%)					
Current	37 (17.7)	29 (16.3)	8 (25.8)	56 (8.8)	93 (11.0)
Former	121 (57.9)	103 (57.9)	18 (58.1)	352 (55.4)	473 (56.0)
Never	47 (22.5)	43 (24.2)	4 (12.9)	211 (33.2)	258 (30.6)
Unknown	1 (0.5)	1 (0.6)	0 (0)	2 (0.3)	3 (0.4)
Systemic therapy, No. (%)					
Chemotherapy	152 (72.7)	131 (74.0)	21 (67.7)	441 (69.4)	593 (70.2)
Immunotherapy	116 (55.5)	99 (55.6)	17 (54.8)	282 (44.4)	398 (47.2)
Targeted therapy	63 (30.1)	56 (31.5)	7 (22.6)	262 (41.3)	325 (38.5)
Metastasis-directed therapy, No. (%)					
SBRT	87 (41.6)	71 (39.9)	16 (51.6)	187 (29.4)	274 (32.5)
SBRT or SRS	123 (58.9)	105 (59.0)	18 (58.1)	237 (37.3)	360 (42.7)
No data	5 (2.4)	5 (2.8)	0 (0)	33 (5.2)	38 (4.5)

Abbreviations: NOS, not otherwise specified; NSCLC, non–small cell lung cancer; SBRT, stereotactic body radiotherapy; SRS, stereotactic radiosurgery.

### Analyzing Genetic Alterations With Metastatic Burden and OS

We first performed a supervised analysis with chi-square testing to compare the frequency of gene alterations between 1-10 and >10 metastases, and 1-4, 5-10, >10 metastases. We calculated the proportions of altered genes within each metastases-number group. Next, we conducted an unsupervised analysis to identify genomic signatures that correlated with outcomes using agglomerative hierarchical clustering. We then evaluated signatures for enrichment in patients with 1-4 and 5-10 metastases. We used Scree plot analysis to identify an optimal number of signatures ensuring that no signature had <10 patients.

For the top 50 altered genes, we compared mutant versus wild-type (WT) for differences in survival within groups of 1-4 metastases or ≥5 metastases. We used Kaplan-Meier log-rank analyses to visualize and compare OS between groups. When clinically significant, we used Cox proportional hazards modeling and subgroup analyses to control for and interpret the effects of systemic therapy category received (chemotherapy, immunotherapy, or targeted agents) and receiving MDT, which was defined as ablative dose radiation with stereotactic body radiotherapy and stereotactic radiosurgery (SRS) in the context of metastatic disease. Finally, we varied cutpoints between 1 and 10 to dichotomize metastasis number and determined the absolute value of the coefficient estimate from a Cox proportional hazards model predicting OS. A heatmap was created using a normal transformation of the estimates standardized by gene. Additional details on methods are provided in Appendix 1.

## RESULTS

### Cohort Characteristics

We identified 844 patients with metastatic NSCLC who met inclusion criteria (Table 1). Seven hundred eighty-nine (93.5%) had de novo metastatic disease, and 55 (6.5%) had recurrent metastatic disease after initial definitive local treatment (Table 1). After imaging review, 209 patients (24.8%) had 1-10 metastases, and 635 patients (75.2%) had >10 metastases. The distribution of the number of metastases is shown in Appendix Table A1.

### Assessing for a Cutpoint That Maximized Difference in OS

**FIG 1.**
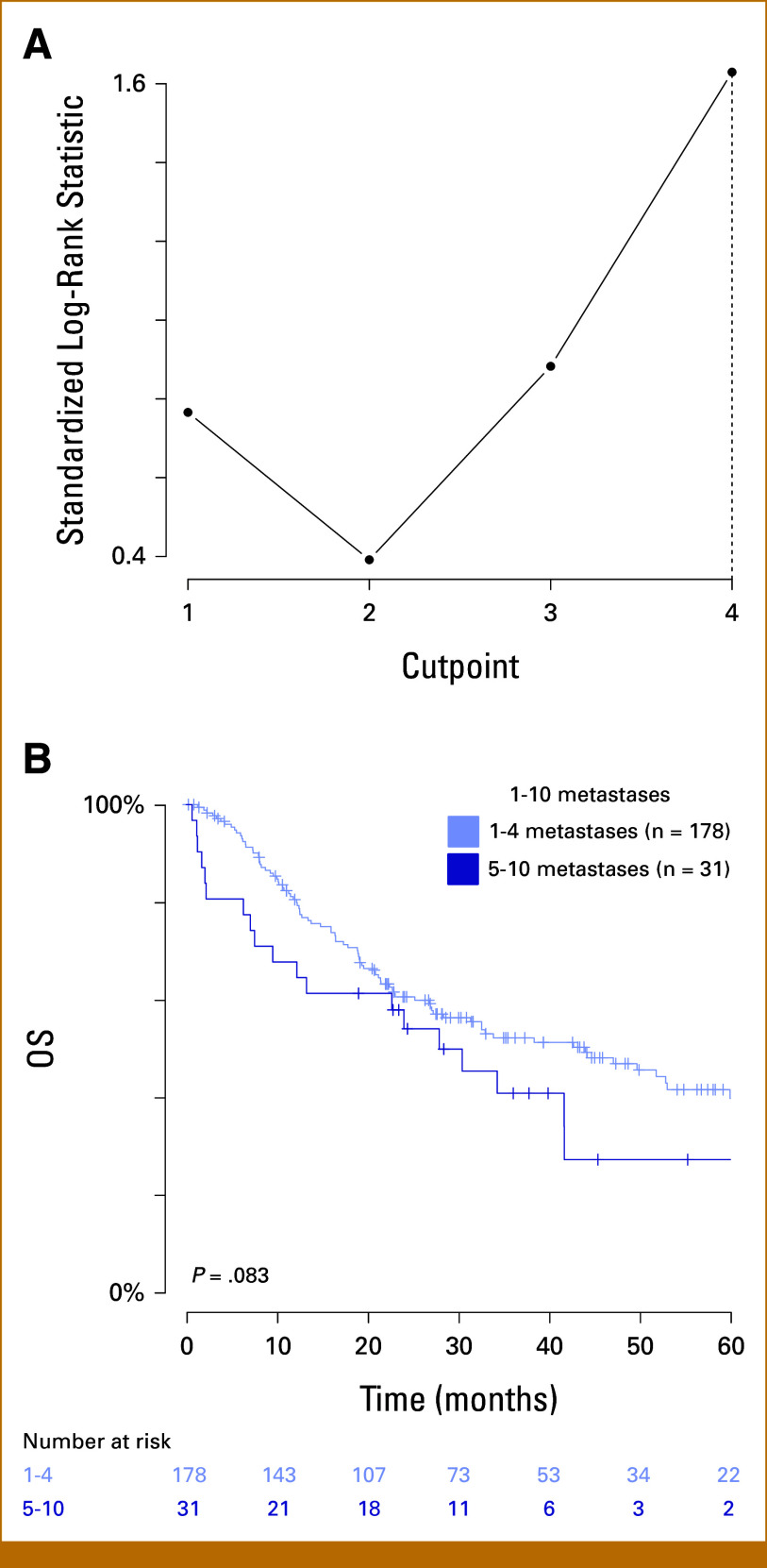
Identifying optimal cutpoint between 1 and 10 metastases to maximize OS. (A) Maximal-rank statistic indicating that four metastases is an optimal cutpoint. (B) Kaplan-Meier curves comparing 1-4 versus 5-10 metastases. The median OS of 44.0 months for patients with 1-4 metastases compared with 27.9 months in those with 5-10 metastases (HR, 0.65 [95% CI, 0.40 to 1.06], log-rank *P* = .081). HR, hazard ratio; OS, overall survival.

**FIG A1.**
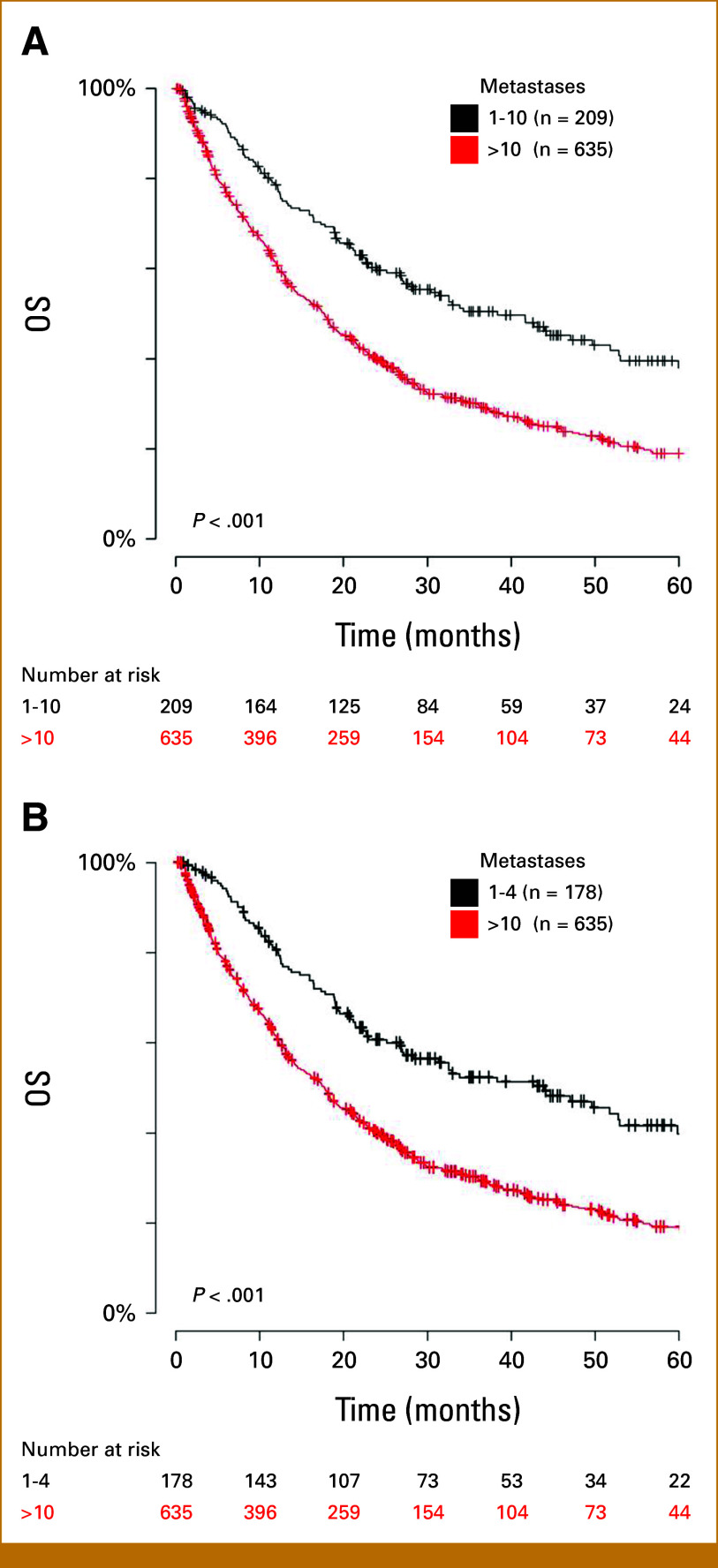
OS comparing patients by number of metastases. (A) 1-10 metastases versus >10 metastases. The median OS was 38.4 months for patients with 1-10 metastases compared with 17.3 months for patients with >10 metastases (HR, 0.56 [95% CI, 0.46 to 0.67], *P* < .01). (B) One to four metastases versus >10 metastases. The median OS was 44.4 months for patients with 1-4 metastases compared with 17.7 months for patients with >10 metastases (HR, 0.72 [95% CI, 0.63 to 0.81], *P* < .01). HR, hazard ratio; OS, overall survival.

**FIG A2.**
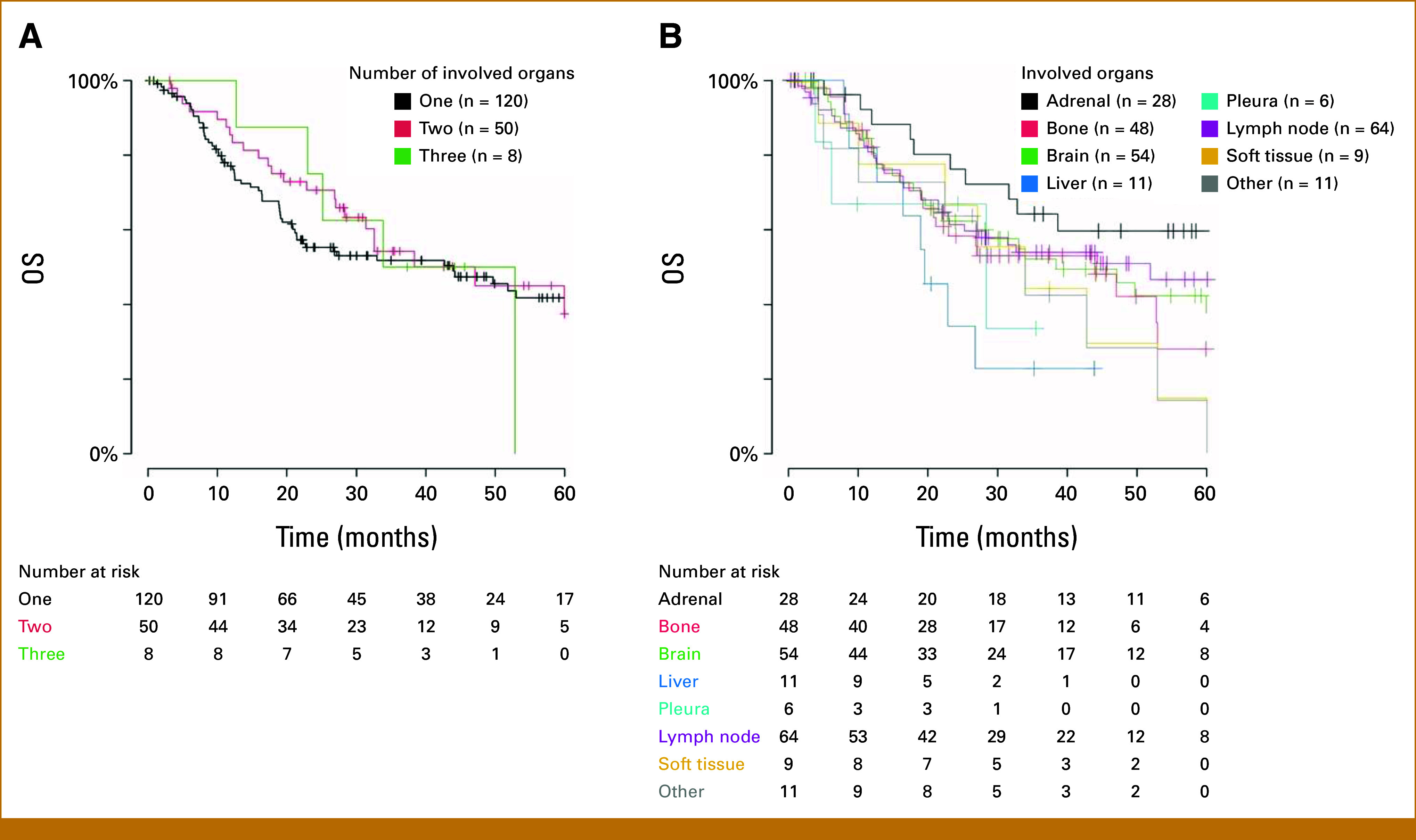
(A) Kaplan-Meier method for OS comparing number of involved organs for patients with 1-10 metastases and (B) OS comparing involved organ type for patients with 1-4 metastases. Because organ involvement was not mutually exclusive, we present a composite Kaplan-Meier plot omitting the uninvolved groups. OS, overall survival.

Median follow-up was 46.2 months, and 66.1% (n = 558) of patients had died by the end of study follow-up. We found that having four or fewer metastases maximized the difference in OS (Fig 1A), with median OS of 44.0 months compared with 27.9 months in those with 5-10 metastases (hazard ratio [HR], 0.65 [95% CI, 0.40 to 1.06], *P* = .101, Fig 1B). In the bootstrap resampling–based analysis, we found that the median cutpoint identified from the 1,000 repetitions was ≤4 metastases, consistent with the cutpoint found by our original data (95% CI, 1 to 5). When only considering patients diagnosed with metastases after January 1, 2016 (patients more likely to receive modern systemic therapy), the cutpoint remained stable at four. We observed improved OS for patients with 1-10 metastases compared with those with >10 metastases (median OS = 38.4 *v* 17.4 months, respectively, HR, 0.55 [95% CI, 0.45 to 0.69], *P* < .001, Appendix Fig A1A). This effect was greater when comparing patients with 1-4 metastases to those with >10 metastases (median OS = 44.0 and 17.7 months, respectively, HR, 0.72 [95% CI, 0.64 to 0.81], *P* < .001, Appendix Fig A1B). Additional outcomes considering organ involvement are described in Appendix Fig A2.

### Correlation of Genetic Alterations With Number of Metastases

**FIG A3.**
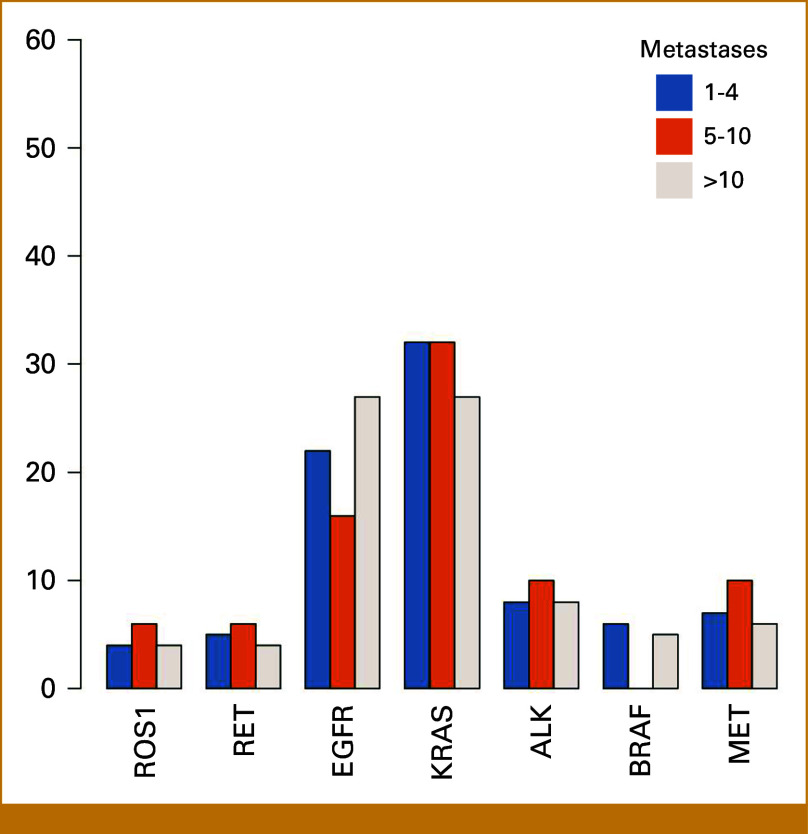
Frequency of major actionable genomic alterations on the basis of number of metastases.

There were no significant differences in the frequency of major actionable genomic alterations in NSCLC (*ROS1*, *RET*, *EGFR*, *KRAS*, *ALK*, *BRAF*, and *MET*) between the cohorts of 1-4, 5-10, and >10 metastases (Appendix Fig A3). *FAT1*, *TP53*, *EPHA5*, *GRIN2A*, *CDKN2A*, *ZFHX3*, *NTRK3*, *EPHA3*, and *PTPRD* were preferentially altered in patients with 1-10 versus >10 metastases (Table 2). *PTPR*, *EPHA5*, *CDKN2A*, *FAT1*, *PTPRD*, *NTRK3*, *ZFHX3. KEAP1*, *SMARCA4*, and *TP53* were more frequently altered in the 5-10 cohort, while *GRIN2A* was more frequently altered in the 1-4 cohort (Table 3).

**TABLE 2.** Genomic Alterations With Significantly Different Frequency Between 1-10 and >10 Cohorts

Gene	1-10, %	>10, %	FDR q-Value
*FAT1*	15.0	5.7	0.001
*TP53*	72.5	57.8	0.003
*EPHA5*	10.6	4.0	0.004
*GRIN2A*	12.6	5.2	0.004
*CDKN2A*	12.1	5.9	0.023
*ZFHX3*	10.1	4.6	0.023
*NTRK3*	9.7	4.4	0.029
*EPHA3*	9.2	4.3	0.036
*PTPRD*	13.0	7.3	0.042

Abbreviation: FDR, false discovery rate.

**TABLE 3.** Genomic Alterations With Significantly Different Frequency Between 1-4, 5-10, and >10 Cohorts

Gene	1-4, %	5-10, %	>10, %	FDR q-Value
*PTPRT*	10.2	22.6	7.0	0.014
*EPHA5*	8.0	25.8	4.0	<0.001
*CDKN2A*	10.8	19.4	5.9	0.012
*FAT1*	13.6	22.6	5.7	<0.001
*PTPRD*	11.9	19.4	7.3	0.034
*NTRK3*	8.0	19.4	4.4	0.004
*ZFHX3*	9.1	16.1	4.6	0.014
*KEAP1*	19.3	35.5	14.9	0.015
*SMARCA4*	13.1	22.6	8.7	0.034
*TP53*	70.5	83.9	57.8	0.002
*GRIN2A*	14.2	3.2	5.2	0.001

Abbreviation: FDR, false discovery rate.

### Patterns of Genetic Alterations and OS

**FIG 2.**
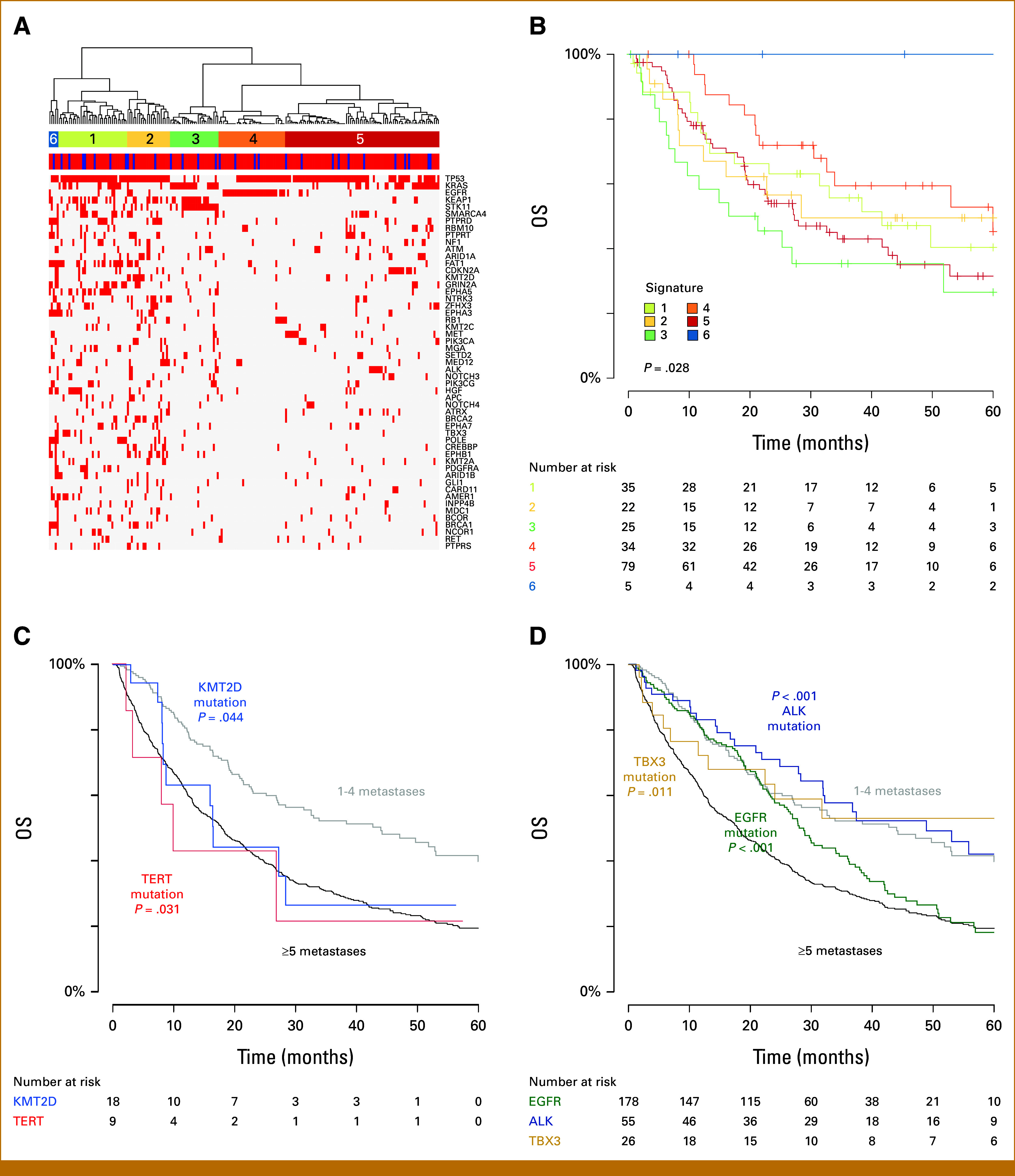
(A) Mutation signatures identified through unsupervised hierarchical clustering. (B) Kaplan-Meier curves showing survival associated with each gene signature. (C) Demonstration of mutations in *KMT2D* and *TERT* conveying inferior OS within 1-4 metastases. (D) Demonstration of *ALK*, *EGFR*, and *TBX3* mutation conveying superior survival within ≥5 metastases. OS, overall survival.

Considering only the patients with 1-10 metastases, we identified six signatures of mutations (Fig 2A). When comparing the signature 3 with all others, there was a significant association with inferior OS (median 18.8 months, log-rank *P* = .028, Fig 2B). The signatures are further detailed in Appendix Table A2.

For patients with 1-4 metastases and the top 50 altered genes, mutations in *TERT* (n = 9) and *KMT2D* (n = 18) were observed to significantly correlate with inferior OS. In this group, mutant *TERT* had a median OS of 10.0 months versus *TERT* WT of 44.0 months (*P* = .031), and mutant *KMT2D* had a median OS of 16.5 months versus *KMT2D* WT of 44.2 months (*P* = .044). Qualitatively, altered *TERT* or *KMT2D* in patients with 1-4 metastases conferred outcomes similar to those with ≥5 metastases (Fig 2C).

For patients with ≥5 metastases, alterations in *EGFR* (median: 28.0 months [mutant] *v* 13.1 months [WT], *P* < .001), *ALK* (median: 48.9 months [mutant] *v* 16.4 months [WT], *P* < .001), and *TBX3* (median: 67.8 months [mutant] *v* 17.7 months [WT], *P* = .011) were associated with improved OS. Qualitatively, *ALK*, *EGFR*, and *TBX3* in patients with ≥5 metastases conferred outcomes similar to those with 1-4 metastases (Fig 2D).

For patients with ≥5 metastases, alterations of *STK11* (median 6.5 months [mutant] *v* 20.5 months [WT], *P* < .001), *KEAP1* (median 5.9 months [mutant] *v* 21.4 months [WT], *P* < .001), *SMARCA4* (median 6.5 months [mutant] *v* 18.6 months [WT], *P* < .001), *KRAS* (median 12.2 months [mutant] *v* 21.6 months [WT], *P* < .001), *ARID2* (median 8.7 months [mutant] *v* 18.2 months [WT], *P* = .012), and *NF1* (median 10.8 months [mutant] *v* 18.4 months [WT], *P* = .020) were associated with inferior OS.

### Analysis Adjusted for MDT

We sought to determine the role of treatment on the associations with molecular alterations described above. First, we assessed the impact of MDT. Most patients with 1-4 metastases and *TERT* mutations did not receive MDT (n = 6), which precluded subgroup analysis. Among *KMT2D*-mutated disease and 1-4 metastases, seven patients received MDT and the associated poor OS was independent of receiving MDT (adjusted *P* = .041). Similarly, the association of *EGFR*, *ALK*, and *TBX3* mutations with superior OS in ≥5 metastases was maintained after adjusting for MDT (adjusted *P* < .001, *P* < .001, and *P* = .005, respectively).

### Analysis Adjusted for Systemic Therapy

Next, we investigated the role of systemic therapy on outcomes for the above alterations, to ensure that variations in standard-of-care systemic therapy did not account for the correlations in outcome on the basis of alteration status. Among *KMT2D*-mutated disease, only one patient received targeted therapy and only one patient did not receive chemotherapy. The poor OS associated with *KMT2D* mutations was independent of immunotherapy use (adjusted *P* = .011). Almost all patients (95.5%) with *EGFR*-mutant disease received targeted therapy, but for the eight patients who did not, median OS was 8.7 months and did not differ by receipt of chemotherapy (*P* = .60). Medianwild OS was not significantly different for patients with *ALK* mutations receiving targeted therapy, although it was nominally higher (median: 55.8 months *v* 28.3 months, *P* = .10). Likewise, chemotherapy and immunotherapy receipt did not significantly discriminate OS in *ALK*-mutated disease, *P* = .99 and *P* = .90, respectively. Finally, most patients with *TBX3* mutations (86.4%) received chemotherapy.

### Metastases Cutpoint on the Basis of Genetic Alterations

**FIG 3.**
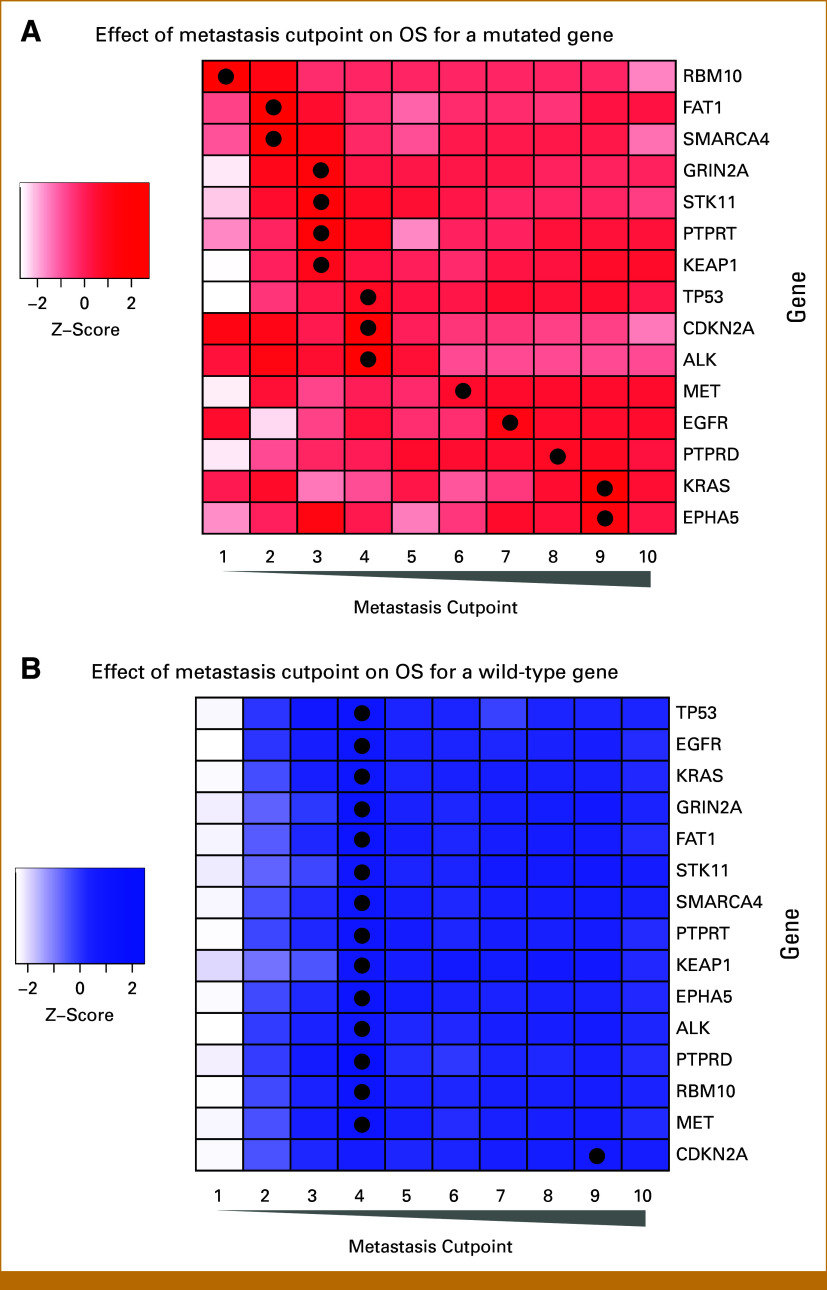
Effect on OS of dichotomizing number of metastases by varied cutpoints, with the darker shades representing stronger associations and a black circle at the number of metastasis with largest effect on OS. (A) All patients with a mutation in a particular gene: For *RBM10*, the largest effects on OS were observed when comparing one versus two or more metastases. For *FAT1* and *SMARCA4*, two or fewer versus three or more showed the largest effect on OS. For *GRIN2A*, *STK11*, *PTPRT*, and *KEAP1*, three or fewer versus four or more showed largest effect on OS. For *TP53*, *CDKN2A*, and *ALK* four or fewer versus five or more showed maximum effect. For *EGFR*, *PTPRD*, *KRAS*, and *EPHA5*, the maximum effect was observed at cutpoints between 5 and 10. (B) All patients who are wild-type for a particular gene: *TP53*, *EGFR*, *KRAS*, *GRIN2A*, *STK11*, *SMARCA4*, *KEAP1*, *EPHA5*, *ALK*, *PTPRD*, *RBM10*, and *MET*, when WT, had a maximum effect when considering four or fewer metastasis compared with five or more, whereas a higher cutpoint was associated with the maximal OS for *CDKN2A*. OS, overall survival; WT, wild-type.

**FIG A4.**
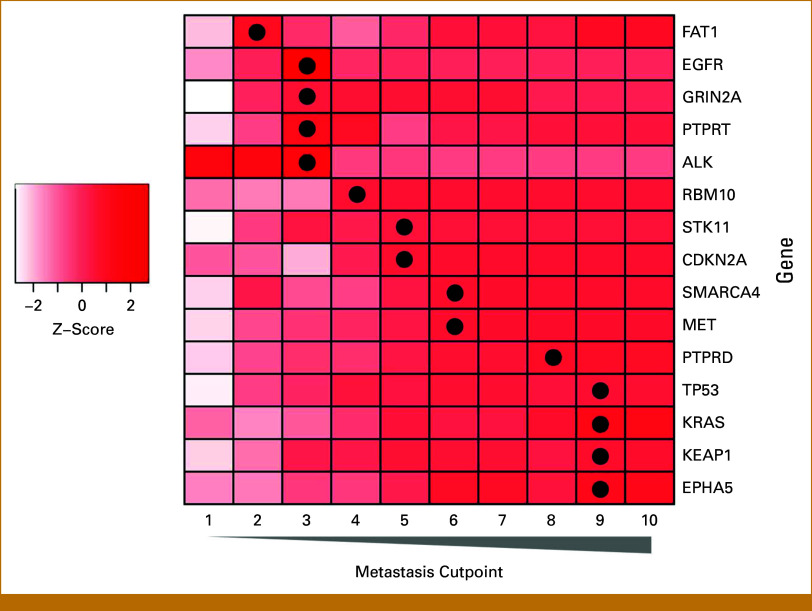
Effect on OS of dichotomizing number of metastases by varied cutpoints, with the darker shades representing stronger associations only for patients receiving immunotherapy. OS, overall survival.

The optimal cutpoint of metastasis number that maximized difference in OS was observed to vary on the basis of the mutation status. Figure 3A demonstrates the strength of association between OS and number of metastases on the basis of a given genetic alteration. For example, for *FAT1* and *SMARCA4*, two or fewer versus three or more showed the largest effect on OS, whereas for *EGFR*, *PTPRD*, *KRAS*, and *EPHA5*, the maximum effect was observed at cutpoints between 5 and 10. Likewise, when analyzing the presence of WT for a given gene, *CDKN2A* had a higher cutpoint of nine compared with WT of the remaining genes (Fig 3B). The impact of immunotherapy on the cutpoint for altered genes is reported in Appendix Fig A4.

## DISCUSSION

In this study, we leveraged a large cohort of patients with metastatic NSCLC with enumerated metastatic sites and comprehensive genomic profiling to assess how multimodal data can elucidate disease trajectories. As expected, having greater than 10 metastases was associated with significantly worse OS. For patients with 1-10 metastases, four metastases was the cutpoint suggested to maximize the difference in OS. Certain genes were more frequently altered in patients with 1-10 metastases, but several were enriched in patients with 5-10 metastases with inferior outcomes. *EGFR*, *ALK*, and *TBX3* alterations were associated with improved OS, even with high metastatic burden, while alterations in *KMT2D* and *TERT* associated with poor outcomes even with low tumor burden. Finally, by demonstrating that the presence of specific alterations may alter the number of metastases that differentiates OS, we demonstrate proof of principle that integrating clinical information with genomic characterization can improve prognostic classification.

Determining the relevant threshold for oligometastatic NSCLC (OMD) is important for designing trials, estimating accrual, and determining inclusion criteria, which have typically been three or five metastases in studies suggesting benefit of consolidative MDT.^9-12^ Consensus statements have also concluded that MDT with radiotherapy can be considered for 1-5 safely treatable metastases,^19^ but investigating MDT to up to 10 metastases remains of interest.^16^ Despite the large number of studies reporting on OMD, our understanding of its true incidence remains surprisingly limited. Estimates range broadly, from 16% to 54%,^14,23-26^ but challenges arise from different definitions of OMD.^19,27^ On the basis of metastasis number alone, we observed that four metastases appeared to maximally separate OS difference, which represented 21.1% of our patients. Although the difference above and below this cutpoint was not significantly different, we believe this signal should be explored further and validated in other cohorts. These data also support the existing approach of 3-5 metastases in trials investigating MDT.

Nevertheless, selecting patients for trial inclusion by number of metastases at a static time point has limitations because of significant heterogeneity of workup and disease course.^27,28^ However, a biological influence on outcomes is supported by studies showing variations in microRNA expression correlating with progression to polymetastatic disease,^29,30^ which may emanate from heterogeneity of genomic mutations.^31^ Incorporation of genomics for patient selection may identify patients with metastatic NSCLC with better prognosis who should be investigated for impact of MDT on outcomes.^17,32,33^ Furthermore, identifying additional metrics of tumor burden may augment multimodal patient selection methods.^34^

In our series, there was no significant difference in the frequency of major actionable genomic alterations between the groups, but patients with 1-10 metastases were more likely to have alterations in certain genes. Interestingly, certain alterations were more likely seen in the intermediate group of 5-10 metastases, which supports heterogeneity even within the 1-10 metastases range. Possible mechanistic reasons for this difference include observations that alterations affecting cell adhesion and junctional organization can impact early-metastatic spread.^35^ Other mechanisms include induction of a hybrid epithelial-to-mesenchymal transition phenotype (FAT1), promoting invasion and migration while suppressing natural killer cell cytotoxicity (EPHA5), and hyperactivation of prometastatic factors (PTPR family).^36-38^ For patients with five or more metastases, alterations in *STK11*, *SMARCA4*, *KRAS*, and *KEAP1*, *ARID*, and *NF1* conferred a poor prognosis, but did not in patients with 1-4 metastases. For patients with 1-4 metastases, *KMT2D* and *TERT* alterations were associated with worse survival, but this association was not present in patients with >5 metastases. This suggests that an alteration's impact on survival can correlate with extent of metastases. *KMT2D* has been shown to act as an epigenetic tumor suppressor in lung cancer through impact on super-enhancers and glycolytic genes.^39^ Activating mutations in *TERT* promotor result in increased telomerase activity and are associated with inferior prognosis.^40,41^ Presence of these alterations in patients with a low volume of metastases may suggest that systemic therapy is preferred over MDT. We further showed that mutation signatures can correlate with outcomes. Signature 3 had poor outcomes, likely because of primary characterization of low tumor mutational burden (TMB) and *KEAP1*/*STK11* alterations, which are both known to have inferior response to immunotherapy.^42,43^

Patients with >5 metastases and mutations in *ALK*, *EGFR*, and *TBX3* had improved survival compared with wild-type in patients with the same extent of metastatic disease. The improved outcomes in patients with *ALK* and *EGFR* alterations are expected and reflect effective US Food and Drug Administration (FDA)–approved targeted therapy for driver mutations of oncogenes.^44^ This supports the observation that presence of certain genetic alterations may drive outcomes more than extent of metastases. Overexpression of *TBX3* is associated with epithelial cancers through impact on proliferation, invasion, and metastases, but further study is needed to understand the impact in lung cancer.^45^ Given the favorable overall outcomes, patients harboring these alterations may be good candidates for MDT at disease progression or in the consolidation setting.

Finally, although having four or fewer metastases maximized OS in the entire cohort, the presence of specific mutations resulted in variation of that cutpoint. The cutpoint decreased when genes such as *SMARCA4*, *STK11*, and *KEAP1* were altered, suggesting more aggressive disease at a lower metastatic threshold. Prior studies have suggested that these mutations may independently confer a poor prognosis, possibly due to conferred therapy resistance or underlying co-occurring mutations.^46,47^ For patients with an *EGFR* mutation, clinical behavior is most distinct when comparing patients with 1-7 versus >7 metastases. This higher cutpoint, while likely representative of an actionable target, suggests considering a higher volume of disease when evaluating for MDT. Overall, the impact of mutations on the threshold suggests that the underlying biology influences prognosis.

We believe that the genetic profile of tumors should guide using a higher or lower cutpoint when considering MDT on the basis of observed differences in prognosis. This can guide the relative aggressiveness of trials using local therapy. If integrated into practice, the selection of sites to biopsy for sequencing must be practical and safe. For our analyses, sequenced sites included primary and metastatic locations, with primary tumor or locoregional nodal tissue prioritized per institutional practice. Although this may raise concern for heterogeneity in sequencing results, the TRACERx study observed limited divergence in genomic alterations from independent sampling of primary and metastatic sites.^48^ Additionally, the tissue was all obtained at the time of metastatic disease diagnosis, which represents a decision time point for therapy. As a result, we believe these data represent a real-world integration of genomic sequencing that can practically assess prognosis and ultimately inform clinical trial selection.

Our study has several limitations. Given our identified cutpoint for OS was not statistically significant, there is risk of type I error. External validation is needed to confirm the cutpoint is robust and that outcomes and genomic correlations are generalizable. However, our tertiary referral center involves multiple regional community sites, which broadens the applicability of these findings. Additionally, given the size of our cohort and lack of other large data sets of metastatic NSCLC with enumerated metastases, we instead assessed for internal validation with data-splitting, acknowledging the limitations.^49^ Our findings are also limited by lack of granular treatment data after metastasis diagnosis. Although this could confound survival estimates, we believe the outcomes apply to a real-world setting. Finally, a limitation of this analysis is that we used a targeted exome panel (MSK-IMPACT) that includes variants of unknown significance. Although this platform performs targeted sequencing for known cancer-related genes, the consequence of a specific alteration requires additional study, especially for previously unknown alterations. However, the focus of the current analysis is to evaluate how the presence of alterations may have prognostic implications. Although the associations do not imply a causative relationship between observed mutations and metastases count, they may be of utility in assessing trajectory of disease.

In conclusion, although four metastases was the cutpoint that maximally differentiated OS in metastatic NSCLC, genetic alterations may influence patient outcomes and modify this cutpoint. Improvement of selection criteria for optimal candidates for MDT is needed. Our data, while hypothesis-generating, suggest that future clinical studies investigating MDT could generate inclusion criteria by combining both genetic and imaging information. Effective implementation of a multimodal and personalized definition of OMD could improve patient selection and optimize treatment options for better outcomes.

## Data Availability

A data sharing statement provided by the authors is available with this article at DOI https://doi.org/10.1200/PO-25-00597.

## APPENDIX 1. SUPPLEMENTARY MATERIALS

### Supplemental Methods

Tumors or involved nodal stations were counted by both reviewing clinical radiology reports and direct image review. We delineated the extent of disease by the number of discrete metastases, not organs involved (eg, three liver metastases were enumerated as 3). Involved metastatic nodal levels were counted as one tumor to correspond with a clinically targetable region during metastasis-directed therapy (MDT), consistent with prior randomized analyses. The presence of effusions, leptomeningeal disease, and other innumerable metastases classified patients as having >10 metastases as they represent a nontargetable treatment area for MDT. Cases with more than 10 discrete metastases or reports indicating extensive metastases were categorized as >10.

Initially, N.A.W. used natural language processing to sort patients into extensive or limited metastases based solely on radiology report text. To do so, NAW looked for the keywords “multiple,” “many,” “numerous,” “widespread,” “innumerable,” “multilevel,” “multifocal,” and “nodules.” NAW initially checked approximately 15% of patients and completed direct image review. Next, research assistants built a RedCap database and manually entered every patient into the database using only the radiology reports. After every 50 cases were entered, N.A.W. and D.R.G. checked the imaging of 10 random patients to ensure data fidelity. Finally, N.A.W. and N.P.M. rechecked radiology reports and direct image review for all patients determined to have fewer than 10 metastases to ensure the lesion enumeration was correct.

We selected 10 metastases as the upper limit as ongoing trials in the oligometastatic space have suggested that up to 10 metastases can be safely and effectively treated with local therapy. Any analysis considering number of metastases was only completed on the fewer than 10 group.

MSK-IMPACT was used for identifying altered genes. MSK-IMPACT is a US Food and Drug Administration–authorized next-generation sequencing panel that performs targeted tumor sequencing with paired tumor and normal tissue. It is a hybridization-based assay that can detect somatic alterations including mutations, copy-number alterations, and structural rearrangements in coding exons of cancer-associated genes. Panels used during the study time period ranged from 341 to 468 genes. The desparsification of mutation data followed previously published methods and involved normalizing the number of alterations per gene by the gene length and accounting for patient-specific mutation burden.

For estimates of overall survival (OS), we obtained either the date of death or date of last clinical follow-up and calculated OS from the metastasis diagnosis date. Patients who were alive were censored at the last clinical follow-up date.

For the cutpoint analysis, we used maximally selected rank statistics. For each cutpoint between 1 and 10 metastases, we computed a two-sample linear rank statistic to test the hypothesis of independence. We then selected a cutpoint that maximized separation in OS between the patients below versus above the cutpoint.

We obtained systemic therapy and MDT data through MSK cBioPortal and the MSK-CHORD databases. Systemic therapy was coded as binary (yes/no) for each patient if received and separated into chemotherapy, targeted therapy, immunotherapy, biologic agents, and investigational drugs. Dates of receipt were not available. MDT was limited to radiotherapy. Radiotherapy courses were indicated relative to MSK-IMPACT biopsy date and included planned dose and fractionation as well as a radiation plan name. Time from metastasis diagnosis to radiotherapy was calculated, and only radiotherapy courses 30 days before metastasis diagnosis and after diagnosis were considered. Common stereotactic radiotherapy dosing regimens (ie, 18 Gy/1fx, 20 Gy/1fx, 27 Gy/3fx, 30 Gy/3fx, 30 Gy/5fx) were identified. Patients in MSK-CHORD who had no radiation data at MSK were assumed to not have had any MDT. Dose and radiation plan name were used to determine stereotactic radiotherapy of brain metastases (eg, stereotactic radiosurgery).

Within 1-4 metastases, we also assessed OS on the basis of the number of metastases, number of organs involved, and which organs were involved. Statistical analysis was performed using *R* software (R-4.1.1). Unless otherwise noted, all references to statistical significance correspond to an α = .05 significance level. To account for multiple hypothesis testing in gene frequency comparisons, we used an FDR q = 0.05 threshold.

### Supplemental Results

In the category of 1-4 metastases, we did not observe differences in OS on the basis of number of organs involved (1 *v* 2-3, *P* = .600, Appendix Fig A2A). With respect to organotropism, when compared with all patients with 1-4 metastases without the observed organ involvement, patients with liver metastases (n = 11) had a shorter median OS of 19.6 months (*P* = .07; Appendix Fig A2B), whereas patients with adrenal metastases (n = 28) had longer median OS of 61.6 months (*P* = .08; Appendix Fig A2B). Patients with metastases in nonthoracic lymph nodes (n = 64, median OS 51.8 months, *P* = .40), brain (n = 54, median OS 38.4 months, *P* = .99), and bone (n = 48, median OS 44.0 months, *P* = .40) did not show a difference in OS. In a combined model for 1-10 metastases comparing the relationship with OS of number of metastases and number of involved organs, 1-4 metastases were associated with borderline significant OS (*P* = .074), while involvement of more than one organ was not (*P* = .563).

### Immunotherapy Analysis

Because STK11, SMARCA4, and KEAP1 have been associated with response to immunotherapy, we specifically tested how the observed effects related to having received immunotherapy. In the subset of patients with >5 metastases who received immunotherapy (n = 299), *STK11* (median 8.9 months [mutant] *v* 20.5 months [WT], *P* < .001) and *KEAP1* (median 10.6 months [mutant] *v* 21.2 months [WT], *P* < .001) mutations were still significantly associated with poor OS. *SMARCA4* trended in the same direction but was not statistically significant (median 11.2 months [mutant] *v* 18.8 months [WT], *P* = .20).

Finally, we considered how the cutpoint may vary with mutation status within patients who received immunotherapy. Changes in estimated cutpoint were observed for EGFR, ALK, CDKN2A, TP53, KEAP1, STK11, and SMARCA4. Given the known associations with KEAP1, STK11, SMARCA4, and immunotherapy, this analysis suggests that even higher thresholds for considering MDT may be possible if patients have systemic therapy options.

**TABLE A1.** Distribution of the Observed Number of Metastases

Number of Metastases	No. of Patients (N = 844)	%	Cumulative %
1	80	9.5	9.5
2	48	5.7	15.2
3	24	2.8	18.0
4	26	3.1	21.1
5	11	1.3	22.4
6	5	0.6	23.0
7	5	0.6	23.6
8	6	0.7	24.3
9	1	0.1	24.4
10	3	0.4	24.8
11-51	635	75.2	100.0

**TABLE A2.** Mutation Signatures Observed Among 1-10 Metastases

Signature	Characterization	No.	OS, Median (months)	TMB %, Median (IQR)
1	High TMB with *TP53* alterations, few *EGFR* alterations, with *KRAS* or *KEAP1* or *GRIN2A* or *EPHA5* or *SMARCA4* or *FAT1* alterations	35	41.7	12.0 (8.3-15.2)
2	High TMB, *TP53* alterations, few *EGFR* alterations, with *KEAP1* or *NTRK3* or *EPHB1* or *KMT2D* alterations	22	28.4	18.3 (11.5-25.2)
3	Low TMB with few *TP53* alterations, and *KEAP1*/*STK11* alterations with *KRAS* alterations	25	18.8	5.3 (3.3-7.3)
4	Low TMB with *TP53* and *EGFR* alterations	34	60.0	2.7 (2.0-4.0)
5	Low TMB, many *TP53* alterations, few *EGFR* alterations, and mutually exclusive alterations in *KRAS*, *ARID1A*, *CDKN2A*, *ALK*, *MET*, *NOTCH4*, *PIK3CA*, or *AFHX3*	79	27.2	5.3 (4.0-7.3)
6	High TMB and otherwise uncategorized	5	61.6	40.0 (22.7-48.7)

Abbreviations: OS, overall survival; TMB, tumor mutational burden.
